# Human Chorionic Gonadotropin vs. Magnesium Sulfate (MgSO4) as a Tocolytic Agent: A Randomized Controlled Trial

**DOI:** 10.7759/cureus.96415

**Published:** 2025-11-09

**Authors:** Hafiza Faiza Mushtaq, Qamar Iqbal, Amna Shaukat, Mishal Maqbool, Fariha

**Affiliations:** 1 Obstetrics and Gynaecology, Mother and Child Hospital, Shaheed Zulfiqar Ali Bhutto Medical University, Islamabad, PAK; 2 Obstetrics and Gynaecology, Federal Government Polyclinic Hospital, Islamabad, PAK

**Keywords:** beta human, comparative study, magnesium sulphate, preterm labour, tocolysis

## Abstract

Objective

To compare the efficacy of β-human chorionic gonadotropin (β-HCG) with magnesium sulfate (MgSO_4_) as a tocolytic agent in preterm labor.

Methodology

This randomized controlled trial (NCT05828966) was conducted in the Department of Gynecology and Obstetrics, Shaheed Zulfiqar Ali Bhutto Medical University (SZABMU), Pakistan Institute of Medical Sciences, Islamabad, Pakistan, from February 15 to December 20, 2023 (11 months). All patients fulfilling the inclusion criteria were enrolled. A total of 70 patients with established preterm contractions and labor were included in the study. All patients were divided into groups A and B via a lottery method. HCG was given in Group A in a dose of 5,000 units intramuscular injection, followed by a drip of 10,000 units in 500 mL of dextrose 5%, at the rate of 20 drops per minute. Patients were given 4 g of MgSO_4_ in Group B intravenously as a continuous dose, followed by 2 g/hour till uterine quiescence is achieved. Half-hourly assessment of uterine contractions, maternal vital signs, and fetal heart rate monitoring was done.

Results

In this study, we enrolled 81 females. There was no significant difference between the overall mean age (25.21 ± 2.992) years, parity (1.49 ± 0.974), and gestational age (31 ± 1.445) years in both groups. HCG had higher patient satisfaction and a lower rate of tocolysis failure within 48 hours compared to MgSO_4_ (*P*-value < 0.001). The HCG group also had a lower rate of preterm labor and lower rates of side effects compared to the MgSO_4_ group (*P*-value = 0.010). However, cesarean section rates were higher in the MgSO_4_ group (*P*-value = 0.025).

Conclusions

HCG treatment is more effective in preventing preterm labor within the critical first 48 hours of pregnancy. As compared to MgSO_4_, HCG exhibits potent tocolysis with the least maternal and neonatal side effects.

## Introduction

Preterm labor is defined as regular uterine contractions associated with cervical changes resulting in changes in the cervix (effacement and dilatation) that start before 37 weeks of pregnancy [1]. Preterm labor accounts for only 10% of all labor, yet 70% of infant deaths are attributed to premature infants. This metric contributes to the assessment of health conditions around the world. Several factors can increase the risk of preterm labor, including a history of previous preterm labor, a short cervix, short intervals between pregnancies, a teenage pregnancy, a history of specific types of surgeries on the uterus or cervix, and certain pregnancy complications, such as multiple pregnancies and vaginal bleeding. Lifestyle factors such as low pre-pregnancy weight, smoking during pregnancy, and substance abuse during pregnancy can also contribute to preterm labor. Clinically, preterm labor can be diagnosed only when changes in the cervix are detected during a pelvic examination, along with monitoring of contractions. A transvaginal ultrasound can be utilized to determine the length of the cervix. Tocolytics delay delivery for a short period (up to 48 hours), allowing time for administration of corticosteroids, magnesium sulfate (MgSO_4_), or for transfer to a hospital that specializes in preterm infant care [1,2]. 

The most commonly used tocolytic agents include nifedipine, magnesium sulfate (MgSO_4_), β-agonists (such as salbutamol), atosiban, and β-human chorionic gonadotropin (β-HCG). β-agonists are rarely used nowadays. MgSO_4_ is preferred when both neuroprotection and tocolysis are required, while β-HCG remains less common due to its high cost in low- and middle-income countries. Salbutamol leads to numerous cardiovascular complications, such as ventricular arrhythmia, hypertension, and hyperglycemia. In contrast, atosiban is expensive and not available in Pakistan. β-HCG is a heterodimeric glycoprotein produced primarily in the placenta and has multiple endocrinological, paracrine, and immunoregulatory functions [3]. β-HCG plays a significant role in maintaining early pregnancy. Reports have suggested that β-HCG keeps the uterus quiescent in the third trimester. A concentration-dependent inhibitory effect of β-HCG can be observed on human myometrial contractions [4]. A recent study suggests that β-HCG plays an endogenous tocolytic role during pregnancy. A significant decrease in serum β-HCG level was found two to three weeks before spontaneous labor began. As a result, the uterine muscle might become more contractile and gradually initiate labor [5].

β-HCG reached its peak concentration approximately six hours after intramuscular injection. It was primarily distributed to the gonads. There was a biphasic decline in blood concentration, with a half-life of 6 to 11 hours and 23 to 38 hours, respectively. The majority of an intramuscular dose was excreted in the urine within 24 hours [6,7]. Preterm labor is associated with a significant increase in neonatal morbidity and mortality. Tocolytic agents were commonly used to delay delivery and inhibit contractions of the uterus. Although both MgSO_4_ and β-HCG have been used as tocolytic agents, comparative studies have not been conducted on their effectiveness to date [8]. In this study, MgSO_4_ was compared with β-HCG as tocolytic agents in preterm labor to gather robust scientific evidence to guide clinical decision-making.

## Materials and methods

This randomized controlled trial (RCT) was registered on clinicaltrials.gov (NCT05828966) [9]. Participants in this study provided written informed consent between February and December 2023. This RCT was conducted at the Department of Gynecology and Obstetrics, Mother and Child Hospital (MCH), Shaheed Zulfiqar Ali Bhutto Medical University (SZABMU), Islamabad, Pakistan. Inclusion criteria included individuals aged 20 to 40 years who experienced four uterine contractions per 20 minutes or eight contractions per hour, had singleton pregnancies under 37 weeks of gestation, intact membranes, cervical dilatation less than 3 cm, and cervical effacement less than 80%. Those with cervical dilatation greater than 3 cm, abnormal vaginal bleeding, premature rupture of membranes, cardiopulmonary compromise, or any fetal or uterine anomaly were excluded.

Eighty-eight (88) outpatients/admitted patients at MCH who met the inclusion criteria were included in the research project. Some patients lost follow-up or shifted to another facility, so a total of 70 patients were included in the study. Using the lottery method, patients were randomized into Groups A and B. The patients were enrolled by non-probability consecutive sampling. In addition to demographics, a first-trimester ultrasound dating scan or the last menstrual period was used to determine gestational age, including fundal height. Before randomization, all eligible patients were provided standard obstetric care according to institutional protocols. On admission, patients were clinically assessed, and baseline investigations were performed, including hemoglobin concentration, urinalysis with microscopy, blood group and Rh typing, and blood sugar estimation. Intravenous (IV) fluids were administered as required to correct dehydration or maintain hydration status, and patients were monitored for uterine activity and fetal well-being. Each patient was re-examined following a period of rest and hydration to exclude false labor or contractions secondary to dehydration before confirming eligibility for randomization. This step ensured that only patients with truly threatened preterm labor were included, thereby improving diagnostic accuracy and minimizing unnecessary exposure to tocolytic therapy. Two grams of intravenous ceftriaxone was given stat, followed by doses every six hours to prevent streptococcal infection in neonates.

**Figure 1:**
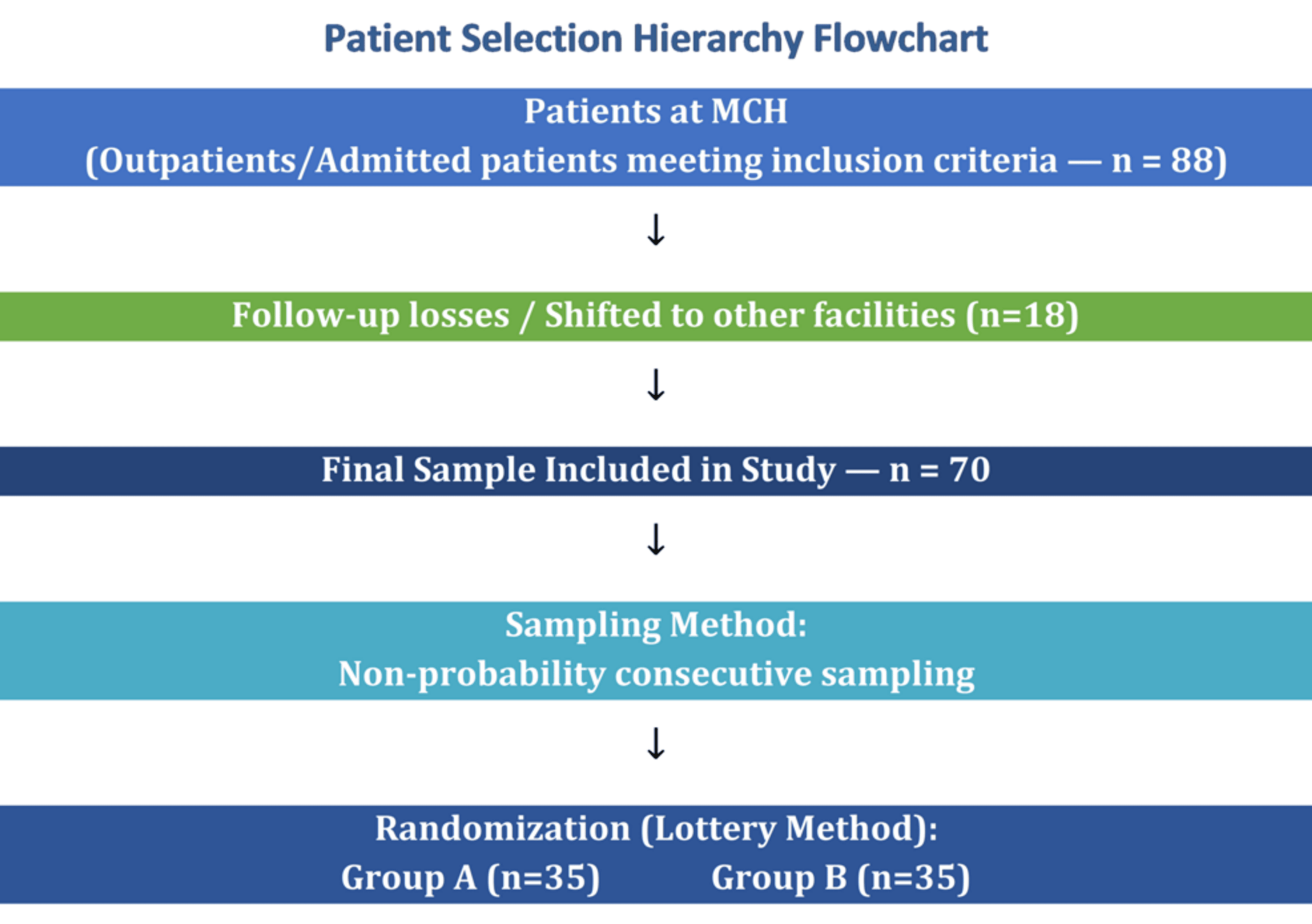
Patient selection hierarchy flowchart. Generated via online software gamma.com.

Five thousand units of β-hCG were injected intramuscularly in Group A, followed by an infusion of 10,000 units in 500 mL of 5% dextrose at a rate of 20 drops per minute. Uterine contractions, maternal vital signs, and fetal heart rate were monitored every half hour. In Group B, 4 g of MgSO_4_ were administered intravenously as a loading dose, followed by 2 g/hour until uterine quiescence was achieved. After cessation of uterine contractions and arrest of labor, patients were observed for 24 hours. The tocolysis was considered successful when contractions ceased and delivery was delayed by 48 hours to allow corticosteroids to accelerate fetal lung maturation.

SPSS version 23.0 (IBM Corp., Armonk, NY) was used to analyze the collected data. The t-test was used to compare mean age, parity, and gestational age between the β-HCG group and the MgSO_4_ group. T-tests were used to compare tocolysis achievement times, while the chi-square test was applied to compare patient satisfaction and tocolysis failure rates between the β-HCG and MgSO_4_ groups. The progression rate to preterm delivery within 48 hours was compared, along with the modes of delivery (normal vaginal delivery and cesarean section), as well as the frequency of various side effects (headache, vertigo, nausea, vomiting, low blood pressure, and no side effects). A *P*-value of less than 0.05 was considered significant.

## Results

This study included 70 female participants. There was no significant difference between the overall mean age (25.21 ± 2.992) years, parity (1.49 ± 0.974), and gestational age (31 ± 1.445) years between the two groups (Table 1).

**Table 1:** Comparison of quantitative variables within groups.

	Mean ± SD (Overall cohort)	β-HCG group	MgSO_4_ group	*P*-value
Age (Years)	25.21 ± 2.992	25.26 ± 3.023	25.17 ± 3.005	0.906
Parity	1.49 ± 0.974	1.43 ± 0.940	1.54 ± 1.010	0.627
Gestational age (weeks)	31 ± 1.445	31 ± 1.435	31 ± 1.475	1.00
Tocolysis achieved (hours)	2.593 ± 0.7815	3.029 ± 0.7270	2.157 ± 0.5658	0.001

A total of 25 patients in the β-HCG group and 9 patients in the MgSO_4_ group reported positive satisfaction (*P*-value = 0.001). The rate of tocolysis failure was lower in the β-HCG group (8 out of 35 patients) compared to the MgSO_4_ group (17 out of 35 patients) (*P*-value = 0.025). As shown in Table 2, the rate of progression to preterm delivery within 48 hours was examined, indicating that β-HCG treatment may prevent preterm labor more effectively within this critical period.

**Table 2:** Comparative analysis of β-HCG and MgSO4 treatment in pregnancy. HCG, human chorionic gonadotropin; MgSO_4_, magnesium sulfate

Variables	HCG group (*n *= 35), *n* (%)	MgSO_4 _group (*n* = 35), *n* (%)	*P*-value
Positive patient satisfaction	25 (71.4%)	9 (25.74%)	<0.001
Failure of tocolysis within 48 hours	8 (22.9%)	17 (48.6%)	0.025
Progress to preterm delivery within 48 hours	6 (17.1%)	16 (45.7%)	0.010
Mode of delivery			
Normal vaginal delivery	4 (11.4%)	7 (20.0%)	0.025
Cesarean section	2 (5.7%)	9 (25.7%)	0.022
Maternal side effects			
Headache and vertigo	0 (0.0%)	15 (42.9%)	<0.001
Nausea and vomiting	9 (25.7%)	2 (5.7%)	0.022
Low blood pressure	2 (5.7%)	6 (17.1%)	0.133
No side effects noted	24 (68.6%)	12 (34.8%)	0.004

The MgSO_4 _group exhibited a higher rate of preterm labor (16 out of 17 patients) compared to the β-HCG group (6 out of 8 patients). Within the critical first 48 hours after intervention, β-HCG treatment appeared to reduce preterm delivery risk. There were 4 out of 6 normal vaginal deliveries in the β-HCG group, compared to 7 out of 16 in the MgSO_4 _group. Compared with the β-HCG group, the MgSO_4_ group had a higher cesarean section rate (9 out of 16 patients) (*P*-value = 0.025).

Headache and vertigo were reported exclusively among the MgSO_4_ group patients (*n* = 15), while these symptoms were not reported in the β-HCG group patients. The β-HCG group had a higher rate of nausea and vomiting (*n* = 9), while the MgSO_4 _group (*n *= 2) had a lower rate (*n *= 2). Low blood pressure was observed in both groups, though slightly more prevalent in the MgSO_4_ group (*n *= 6). Nevertheless, there were fewer side effects in the β-HCG group (*n *= 24) than in the MgSO_4_ group (*n *= 12). A significant association was observed between both groups based on side effects (*P*-value = 0.001), as shown in Table 2.

## Discussion

Preterm labor remains a significant challenge in obstetrics, contributing to neonatal morbidity and mortality. Tocolysis, a cornerstone of preterm birth intervention, aims to delay uterine contractions and reduce associated risks, thereby enhancing neonatal outcomes. The congruence in demographic characteristics, encompassing mean age, parity, and gestational age, across both treatment groups underscores the success of randomization and comparability of study participants [10]. This rigorous approach minimizes the potential influence of confounding factors, enhancing the validity of subsequent comparative analyses. This uniform distribution provides a robust foundation for the interpretation and evaluation of the differing outcomes observed between the two groups, as seen in the study conducted by Rao [4]. 

The results demonstrated a higher rate of patient satisfaction in the β-HCG group compared to the MgSO4 group (*P*-value = 0.001). This finding aligns with previous studies conducted by Tsomos et al. [7] and Sae-Lin and Wanitpongpan [11], indicating that patient satisfaction is an important aspect of tocolytic therapy success. Our study unveiled intriguing discrepancies in the effectiveness of β-HCG and MgSO_4_ in achieving tocolysis within the critical 48-hour window. Patients administered β-HCG demonstrated a significantly expedited achievement of tocolysis compared to the MgSO_4_ group [12,13]. This finding suggests that β-HCG might exert a more rapid and potent effect in suppressing uterine contractions, potentially offering an advantage in managing preterm labor within the pivotal time frame. Divergent rates of patient satisfaction and tocolysis failure emerged as compelling outcomes. Patients treated with β-HCG reported a markedly higher rate of positive satisfaction compared to those given MgSO_4_. Tocolysis failure was significantly lower in the β-HCG group (8 out of 35 patients) compared to the MgSO_4_ group (17 out of 35 patients) (*P*-value = 0.025). This suggests that β-HCG may be more effective in maintaining uterine quiescence and delaying preterm labor [3].

Progression to preterm delivery within 48 hours provided further insights, hinting at β-HCG's potential superiority as a tocolytic agent [14]. The lower rate of preterm labor observed in the β-HCG group implies that β-HCG treatment might better curtail the risk of early delivery compared to MgSO_4_. This observation aligns with prior research indicating HCG's inhibitory effects on myometrial contractions, contributing to its potential advantages in managing preterm labor. Similar to the study of Mazaki-Tovi et al. [15], the mode of delivery varied significantly between the two groups, with the β-HCG group having a higher rate of normal vaginal deliveries (4 out of 6 patients) compared to the MgSO_4_ group (7 out of 16 patients). Additionally, the MgSO4 group had a higher cesarean section rate (9 out of 16 patients) (*P*-value = 0.025). These findings suggest a potential impact of the tocolytic agent on the mode of delivery.

In terms of maternal side effects, our study identified a significant association between the two groups regarding side effect incidence and nature. Side effects were more prevalent in the MgSO4 group, with patients reporting headache, vertigo, and nausea/vomiting. Conversely, the β-HCG group experienced fewer side effects overall. This aligns with existing literature indicating that β-HCG may have a more favorable side effect profile compared to MgSO_4_ [16]. A significant association was observed between the two groups based on side effects (*P*-value = 0.001). This underscores the importance of considering not only the efficacy but also the safety and tolerability of tocolytic agents in clinical decision-making [17].

This study provides evidence that β-HCG may be a more effective and well-tolerated tocolytic agent compared to MgSO_4_ in a tertiary care hospital setting. The findings emphasize the potential benefits of incorporating β-HCG into clinical practice for the prevention of preterm labor. Further research, including larger-scale RCTs, is warranted to validate these results and explore the long-term outcomes associated with β-HCG as a tocolytic agent. However, acknowledging the study's limitations, including sample size and dosing variations, future research endeavors, including multicenter trials and mechanistic investigations, are warranted for a comprehensive understanding of β-HCG and MgSO_4_'s distinct impacts on tocolysis and preterm birth management.

## Conclusions

β-HCG was found to be more effective than MgSO₄ in achieving tocolysis within the first 48 hours of preterm labor, with fewer maternal side effects and better obstetric outcomes. Although the study was limited by the absence of body mass index (BMI) and neonatal outcome data, β-HCG appears to be a safer and more effective tocolytic option, warranting further research to confirm its long-term maternal and neonatal benefits.
